# The microbiome of the invertebrate model host *Galleria mellonella* is dominated by *Enterococcus*

**DOI:** 10.1186/s42523-019-0010-6

**Published:** 2019-09-03

**Authors:** Camille Nina Allonsius, Wannes Van Beeck, Ilke De Boeck, Stijn Wittouck, Sarah Lebeer

**Affiliations:** Department of Bioscience Engineering, Research Group Environmental Ecology and Applied Microbiology, University of Antwerp, Groenenborgerlaan 171, B-2020 Antwerp, Belgium

**Keywords:** *Galleria mellonella*, Greater wax moth, Microbiota, *Enterococcus*, Invertebrate host, Low diversity

## Abstract

**Background:**

The popularity of *Galleria mellonella* as invertebrate model is increasing rapidly, because it forms an attractive alternative to study bacterial, fungal and viral infections, toxin biology, and to screen antimicrobial drugs. For a number of vertebrate and invertebrate animal and plant models, it has been established that the commensals present within the microbial communities on various host surfaces will influence the host’s immune and growth development state and the colonization capacity of newly introduced micro-organisms. The microbial communities of *Galleria mellonella* larvae have, however, not yet been well characterized.

**Results:**

In this study, we present the bacterial communities that were found by *16S rRNA* amplicon sequencing on different body sites of *G. mellonella* larvae. These communities showed very little diversity and were mostly dominated by one *Enterococcus* taxon. In addition, we found that the production conditions (as ‘bait’ for fishing or under more controlled ‘research grade’ conditions - with or without hormones and antibiotics) appear to have little impact on the microbiota of the larvae.

**Conclusions:**

Establishment of the simplicity of the microbiota of *G. mellonella* larvae underlines the potential of the larvae as a model host system for microbiome-host interactions.

## Background

The invertebrate model *Galleria mellonella* or greater wax moth has become a popular alternative to rodent models in several research areas, including bacterial and fungal virulence, viral infections, toxin research and antimicrobial drug testing. This is thanks to the lack of ethical constraints, its short life cycle and its simplicity regarding handing and necessary lab equipment [1–3]. Despite the increasing body of literature using *G. mellonella* larvae, not much is known about their commensal microbiota. To the best of our knowledge, only four studies have used high-throughput *16S rRNA* gene sequencing for the characterization of the gut microbiota of the larvae. The focus of these studies was, however, on how *G. mellonella* manages its gut microbiota during metamorphosis, on the influence of antibiotic feeding on alterations in antibiotic resistance gene pool in the gut microbiota, and the effect of particular *Photorhabdus* strains and envenomation by the ectoparasitoid *Habrobracon hebetor* on the gut microbiota [4–7]. The bacterial diversity of different body niches (e.g. gut versus haemolymph) and interindividual variation has not yet been mapped.

The microbial community of the moth might play a major role in the colonization capacity of newly introduced bacterial species and in the immune state and maturation of the larvae. For example, for the model invertebrate *Drosophila melanogaster*, it has been shown that flies with a normal microbiota, existing of *Acetobacter* and *Lactobacillus* species that probably originate from their feed, are less susceptible to certain infections than flies depleted of these predominant members [8, 9]. In addition, the growth and development rate of *D. melanogaster* is shown to be affected by the microbiota [9, 10]. Also in many other invertebrate and vertebrate animal models, even plants, the microbial community will influence the host’s immune state and the colonization capacity of newly introduced micro-organisms. Currently, two different types of larvae of *G. mellonella* are being used in scientific research, i.e. ‘research grade’ larvae that are grown without hormones or antibiotics and the cheaper ‘bait’ or ‘pet food’ larvae, which are grown for fishery, but the consequences on their microbiota have not yet been described.

Mapping the core microbiota of this model animal could improve our interpretation of disease models and studies on toxin research and antimicrobial drug testing. In this study, we aimed to characterize the microbiome present at different body sites of the *G. mellonella* larvae, namely the skin surface, in the fat body, the haemolymph and the faeces (to represent the gastro-intestinal microbiome) of larvae produced under the most commonly used conditions in microbe-host interaction research, namely bait larvae and research-grade larvae.

## Results

### The microbiome of *Galleria mellonella* larvae shows low diversity at each body site and is dominated by *Enterococcus*

**Fig. 1:**
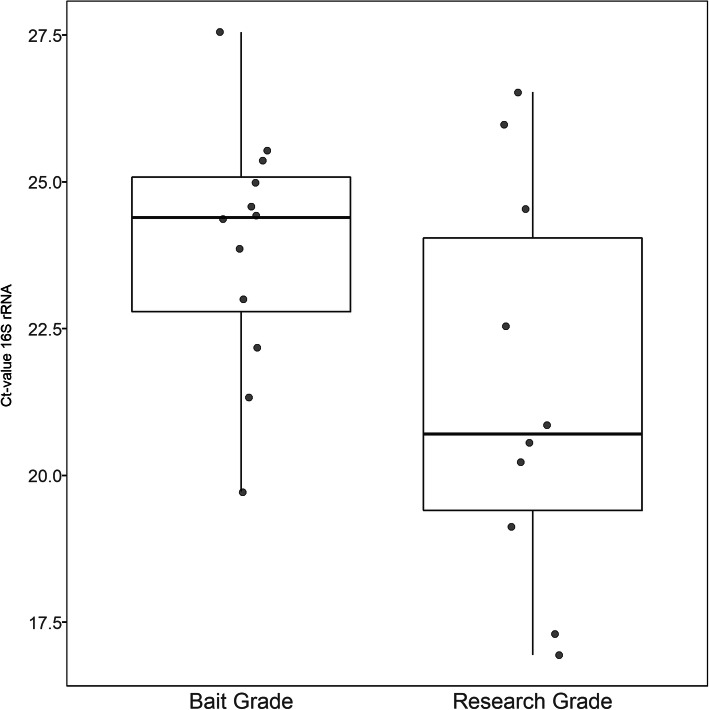
Comparison of bacterial load in the larvae. The difference in bacterial load between the faecal samples of 12 bait and 12 research-grade larvae was estimated by qPCR with general primers for the V4 region of the *16S rRNA* gene

Microbial DNA was obtained from a skin, a faeces, a fat body and a haemolymph sample, from 12 bait and 12 research-grade larvae. To prevent possible cross-contamination, several precautions were taken as outlined in materials and methods. The amplified V4 regions of the 16S rRNA genes of the samples were sequenced by Illumina MiSeq. After denoising the raw sequencing output, the sequenced library consisted out of 6,469,678 reads across 104 samples and 1902 taxa. Before further processing this table, a rigourous quality control was applied as described in Jervis & Bardy [11] as an additional way to account for possible cross contamination. In short, DNA concentrations were estimated by dividing the number of reads by the volume added to the library. Subsequently the relative abundance of each taxon was correlated with this “estimated” DNA concentration (Spearman correlation), and assessed using a fisher exact test. Taxa that were significantly associated with low-concentration samples (negative kit and PCR controls) were removed (threshold, p < e-5). This led to a reduction of taxa from 1633 to 373 taxa. Next, a manual curation of well-known kit contamination species was performed [12]. This lead to a final dataset containing 5,867,192 reads across 96 samples and 343 taxa which was used for downstream analysis including calculations of the diversity between body sites and the generation of overall bacterial community profiles. Of note, we observed significant differences in the concentrations of total extracted DNA across the different body sites studied for both bait and research grade larvae, suggesting a clear difference in bacterial load in these different sites. In general, the haemolymph fluid had the lowest bacterial load, which could be the result of clearance by the immune cells highly abundant in this anatomic site of the larvae [13]. In addition, we also tested whether the rearing conditions influenced the bacterial load in the gut of the larvae by comparing the bacterial DNA concentrations in the faecal samples with qPCR. This comparison showed that the bacterial load in the bait-grade larvae was approximately 5,3 times lower than in the research-grade larvae (Fig. 1, *p*-value = 0.06528), which could be the result of the administration of antibiotics during rearing.

**Fig. 2:**
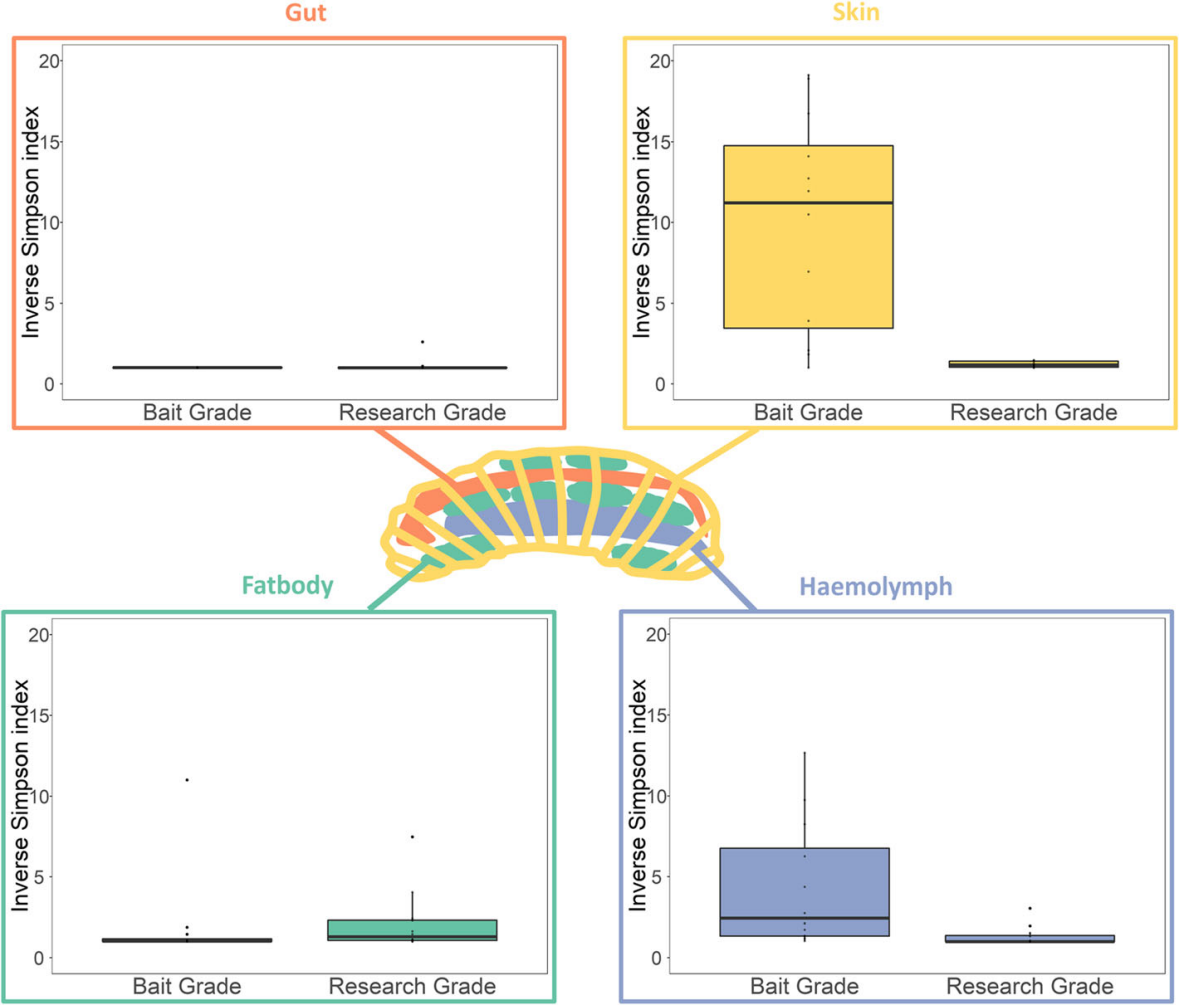
Diversity measures of four different body sites of bait and research-grade *Galleria mellonella* larvae. Observed diversity and Inverse Simpson index for fat body (F), faecal (G), haemolymph (H) and skin samples (S) of 12 bait and 12 research-grade larvae

Both the Observed Diversity index, which only took the presence/absence of bacterial taxa into account, and the Inverse Simpson diversity index, which also took the evenness of the microbial community into account, showed that the overall diversity of the different body sites was very low (Fig. 2). The alpha diversity was the highest for the skin samples (Inverse-simpson index = 9.98) and the haemolymph samples (Inverse-simpson index = 4.39) of the bait grade larvae. Other body sites had an index ranging from 1.01 (faeces) to 2.14 (fatbodies). When comparing body sites within the type of origin, only the larval skin samples showed a significantly higher diversity compared to the fat body (*p* = 0.004) and gut (*p* = 0.001) for the bait larvae and an increasing trend compared to the gut (*p* = 0.056) for the research-grade larvae.

**Fig. 3:**
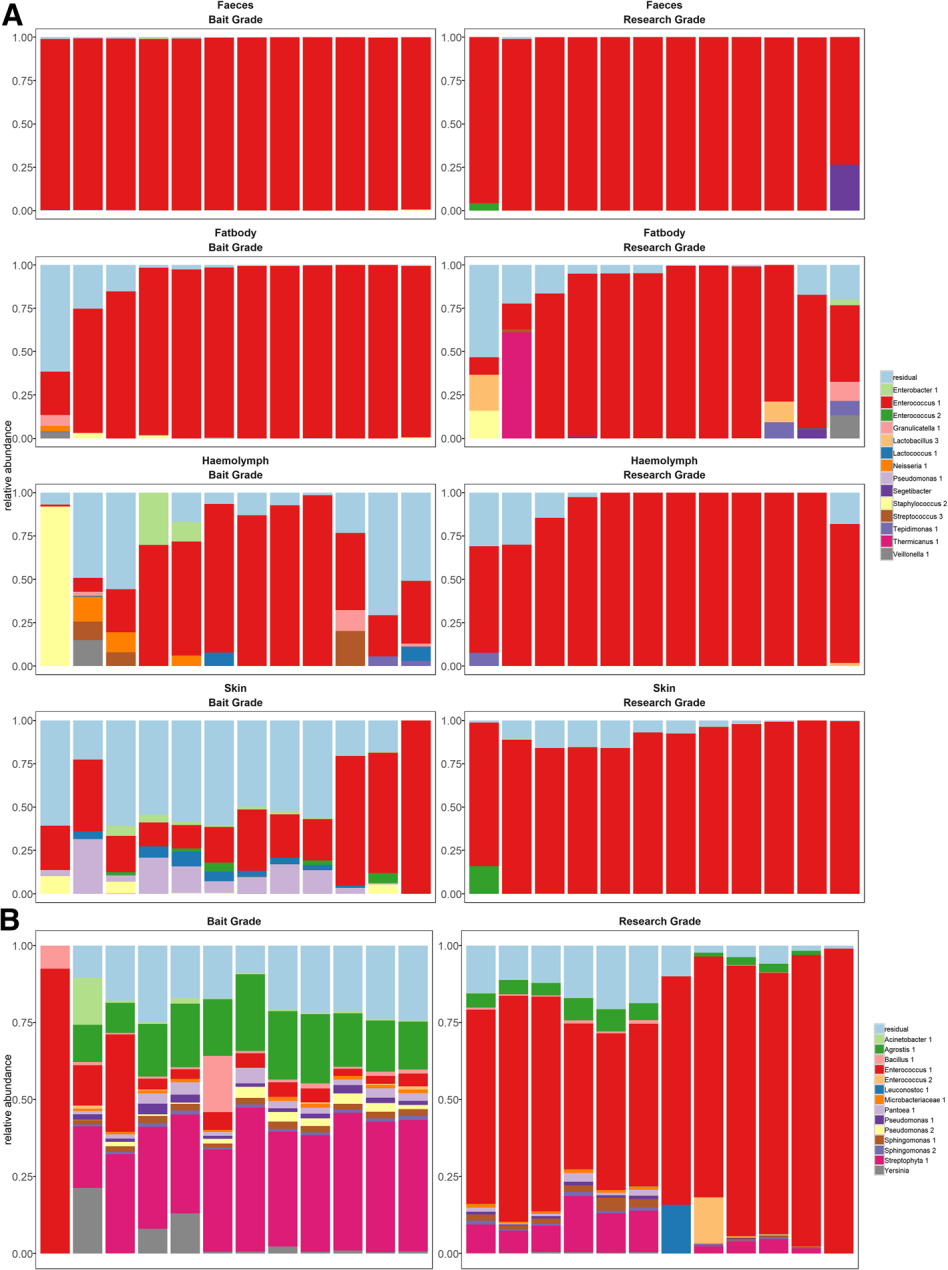
The microbiome composition on different body sites of *G. mellonella* larvae. **a** Comparison of top 14 most abundant amplicon sequence variants (ASVs) in four different body sites (fat body, faecal, haemolymph and skin samples) sampled of 12 bait and 12 research-grade *G. mellonella* larvae. **b** Comparison of the top 14 most abundant bacterial and non-bacterial ASVs on the skin. Community profiles are shown for each sample, depicting the relative abundance of the 14 most abundant ASVs. All others ASVs were grouped under residual

Strikingly, we found the same amplicon sequence variant (ASV), belonging to the genus *Enterococcus* (Fig. 3), in all samples from the different body sites studied here. In all samples, except for the skin and haemolymph of the bait larvae studied, this ASV was also dominant (relative abundance > 50%). Based on further classification at the EZBioCloud (formarly known as EZtaxon) database [14], this ASV could be classified as *Enterococcus gallinarum/saccharolyticus*, a common gut commensal of insect, such as *Drosophila* [15], humans [16] and animals, such as dogs and gulls [17]. To confirm these sequencing results, samples were also plated out on De Man Rogosa Sharpe (MRS) medium and colonies were picked for identification using 16S colony PCR and Sanger sequencing. *Enterococcus* colonies were amongst the most abundant colonies on plate for each of the different body sites. On the skin of the bait larvae however, the community was mainly dominated by two non-bacterial ASVs, classified as a plant-associated mitochondrion (classified as *Streptophyta*) and chloroplast (classified as *Agrostis sp*.) (Fig. 3b). Further investigation of these two genera led to the hypothesis that these two taxa are derived from components of the bedding from the larvae enclosures, in agreement with different rearing conditions of bait larvae coming more into contact with plant material. In addition to the *Enterococcus gallinarum/saccharolyticus*, a few other lactic acid bacteria were found, including *Enterococcus* 2 (*Enterococcus faecium/faecalis,* max 10% relative abundance), *Lactobacillus* and *Leuconostoc*, among other typical gut commensals as *Bifidobacterium* and *Peptoniphilus*. Next to these probably commensal bacteria, we also found some potential pathogenic ASVs, mainly in the bait larvae, such as *Enterobacter*, *Pseudomonas*, *Staphylococcus* and *Streptococcus*.

## Discussion

Our data presented here show that, independent of the body site sampled, *G. mellonella* larvae used for microbiological research are dominated by a single *Enterococcus* taxon, putatively identified as *E. gallinarum* or *E. saccharolyticus* based on the combination of ASV analysis and the curated EzBioCloud 16S rRNA reference database [18], although various other taxa (such as *Staphylococcus, Pseudomonas* and *Enterobacter* species) were also frequently found. The simplicity of the larval microbiota has a number of implications for their role as model host animals. Firstly, as mentioned before, the growth and development rate of model animals are shown to be affected by the microbiota [9, 10]. Therefore, the robust microbial composition of *Galleria mellonella* will enhance experimental reproducibility across scientific institutions. Secondly, results of microbiota studies will be easier to interpret, as there will be little variance introduced by differences in the initial microbiota. It should however be noted that despite the presence of only one dominant ASV, there could still be underlying diversity in the form of different very closely related strains within this ASV. It should also be taken into account that the immune system of the larvae might be skewed by the predominant interaction with enterococci. Of note, long term rearing might have promoted the growth of *Enterococcus gallinarum/saccharolyticus,* abundantly present in the *Galleria* gut to also colonize the other body sites.

Despite the lack of controlled production of *G. mellonella* larvae that are sold as bait, but frequently used for experimental setups, the microbial communities in their gut, haemolymph and fat bodies appeared to be dominated by the same bacterial taxa as research-grade larvae. However, our qPCR did show lower bacterial loads in the bait-grade larvae, possibly resulting from antibiotic administration. It should be taken into account that there is no exact information on hormone or antibiotic administration during rearing available when purchasing bait-grade larvae, while it is guaranteed that the research-grade larvae are grown under very strict rearing conditions. These antibiotics or hormones fed can still be present during your experiment and impact the results. In addition, feeding of antibiotics can enlarge the pool of microbial resistance genes, which can in turn affect experimental outcomes [5].

## Conclusions

Our results corroborate the potential of the *Galleria mellonella* larvae as a model host system due to the relative simple microbiome present on the different body sites and the high comparability in microbiome between individual larvae. In addition, our results indicate that the microbiome of bait larvae can be compared to the more controlled research grade larvae, which also improves the comparability between different experimental set-ups.

## Methods

### Larvae and sample collection

Bait *Galleria mellonella* larvae were purchased from Anaconda reptiles (Kontich, Belgium) and research-grade *Galleria mellonella* larvae, grown without antibiotics and hormones, were a gift from Trularv™ (BioSystems™, Devon, U.K.). Upon arrival, the larvae were stored at 4 °C and used within 7 days. Samples from the skin, faeces, fat body and haemolymph from 12 bait and 12 research-grade larvae were obtained after one day of individual incubation in the dark at 37 °C.

### DNA extraction and bacterial enrichment

The PowerFecal® DNA isolation kit (with Inhibitor Removal Technology®) was used according to the instructions of the manufacturer.

### qPCR

The isolated DNA samples were diluted 10-fold and used to determine bacterial load by qPCR, using the StepOnePlus real time qPCR system (Applied Biosystems) and SYBR® Green chemistry (PowerUp™ Sybr® Green Master Mix, Applied Biosystems). Primers used were 338F (ACTCCTACGGGAGGCAGCAG) and 518R (ATTACCGCGGCTGCTGG) with the followingcycling program: 3 min at 95 °C; 40 cycles of 1 min at 95 °C, 40 s at 56 °C and 40 s at 72 °C [19]. Based on the difference in Ct-value, an estimation of concentration difference can be made based on the 2^–∆∆**Ct**^ method.

### Illumina MiSeq *16S rRNA* amplicon sequencing

The primers used for Illumina MiSeq sequencing were based on the previously described 515F-806R primers and altered for dual-index paired-end sequencing, as described by Kozich et al. (2013) [20, 21]. Briefly, each DNA sample was subjected to dual barcoded PCR, amplifying the V4 region of the *16S rRNA* gene using Phusion High-Fidelity DNA polymerase (New England Biolabs, USA). PCR products were purified by the Agencourt AMPure XP magnetic bead capture kit (Beckman Coulter, Suarlee, Belgium), and quantified using the Qubit® 3.0 fluorometer. The library was prepared by pooling all PCR samples in equimolar concentration and loaded onto a 0.8% agarosegel. The product was purified by gel extraction using the Nucleospin® Gel and PCR clean-up (Machery-Nagel). The purified library concentration was determined with the Qubit® 3.0 fluorometer and diluted to a final concentration of 2 nM. The library was denatured with 0.2 N NaOH (Illumina), diluted to 6 pM and spiked with 10% PhiX control DNA (Illumina). The library was loaded onto the flow cell of the v2 chemistry MiSeq reagent kit (paired-end dual indexing sequencing; 2 × 251 bp kit; Illumina, San Diego, California, USA) on the MiSeq Desktop sequencer (M00984, Illumina) at the Centre of Medical Genetics, University of Antwerp, Belgium.

### Sequence processing and biostatistical analysis

Raw sequencing reads were filtered and denoised using the DADA2 (Divisive Amplicon Denoising Algorithm 2) pipeline (v 1.1.6), as described in [22]. The DADA2 method is a denoising algorithm that infers the set of most specific biological variants (called amplicon sequence variants (ASVs)) that are not the result of sequencing errors. In short, paired reads were filtered by excluding reads with more than two expected errors and reads that contained undetermined bases. Based on a visual inspection of the quality score profiles, trimming was done by removing first 12 nucleotides on forward and reverse strand. Next, DADA error correction was applied using the error model constructed by alternation of sample inference and error rate estimation until convergence. Forward and reverse reads were then merged into contigs. At this point, chimeras were removed. Taxonomic annotation from the kingdom to the genus level was then assigned to the remaining ASVs, making use of the EzBioCloud 16S rRNA reference database (version mtp1.5, update 2018.05). Finally, ASVs classified as Archaea, Eukarya, chloroplasts or mitochondria were removed. Secondly an alternative analysis was performed containing also non-bacterial ASVs.

The resulting ASV table was imported and analysed in R, using the in-house developed package tidyamplicons (www.github.com/swittouck/tidyamplicons), ggplot2 (v 2.1.0) [23], and the vegan package (v 2.3–5) [24]. Quality control was performed as described in the Result section. Observed ASV-richness and the inverse Simpson index calculated on the non-normalized read count data were used as alpha-diversity indices. The relative abundances of the top 14 ASVs were plotted to assess the bacterial community composition.

## Data Availability

Sequencing data are available at the European Nucleotide Archive with the accession number PRJEB31807 (https://www.ebi.ac.uk/ena/data/view/PRJEB31807)*.* Scripts used for generating the figures and performing quality control are available at https://github.com/LebeerLab/Galleria.

## Abbreviations

ASV: Amplicon sequence variant
DADA2: Divisive Amplicon Denoising Algorithm 2
DNA: Deoxyribonucleic acid
MRS: De Man Rogosa Sharpe
PCR: Polymerase chain reaction
rRNA: Ribosomal ribonucleic acid
